# High Expression of CD109 Antigen Regulates the Phenotype of Cancer Stem-Like Cells/Cancer-Initiating Cells in the Novel Epithelioid Sarcoma Cell Line ESX and Is Related to Poor Prognosis of Soft Tissue Sarcoma

**DOI:** 10.1371/journal.pone.0084187

**Published:** 2013-12-20

**Authors:** Makoto Emori, Tomohide Tsukahara, Masaki Murase, Masanobu Kano, Kenji Murata, Akari Takahashi, Terufumi Kubo, Hiroko Asanuma, Kazuyo Yasuda, Vitaly Kochin, Mitsunori Kaya, Satoshi Nagoya, Jun Nishio, Hiroshi Iwasaki, Tomoko Sonoda, Tadashi Hasegawa, Toshihiko Torigoe, Takuro Wada, Toshihiko Yamashita, Noriyuki Sato

**Affiliations:** 1 Department of Orthopedic Surgery, Sapporo Medical University School of Medicine, Chuo-ku, Sapporo, Japan; 2 Department of Pathology, Sapporo Medical University School of Medicine, Chuo-ku, Sapporo, Japan; 3 Department of Surgical Pathology, Sapporo Medical University School of Medicine, Chuo-ku, Sapporo, Japan; 4 Department of Orthopedic Surgery, Fukuoka University School of Medicine, Nanakuma, Jonan Ward, Fukuoka, Japan; 5 Department of Pathology, Fukuoka University School of Medicine, Nanakuma, Jonan Ward, Fukuoka, Japan; 6 Department of Public Health, Sapporo Medical University School of Medicine, Chuo-ku, Sapporo, Japan; Wayne State University School of Medicine, United States of America

## Abstract

Epithelioid sarcoma (ES) is a relatively rare, highly malignant soft tissue sarcoma. The mainstay of treatment is resection or amputation. Currently other therapeutic options available for this disease are limited. Therefore, a novel therapeutic option needs to be developed. In the present study, we established a new human ES cell line (ESX) and analyzed the characteristics of its cancer stem-like cells/cancer-initiating cells (CSCs/CICs) based on ALDH1 activity. We demonstrated that a subpopulation of ESX cells with high ALDH1 activity (ALDH^high^ cells) correlated with enhanced clonogenic ability, sphere-formation ability, and invasiveness *in vitro* and showed higher tumorigenicity *in vivo*. Next, using gene expression profiling, we identified CD109, a GPI-anchored protein upregulated in the ALDH^high^ cells. CD109 mRNA was highly expressed in various sarcoma cell lines, but weakly expressed in normal adult tissues. CD109-positive cells in ESX predominantly formed spheres in culture, whereas siCD109 reduced ALDH1 expression and inhibited the cell proliferation *in vitro*. Subsequently, we evaluated the expression of CD109 protein in 80 clinical specimens of soft tissue sarcoma. We found a strong correlation between CD109 protein expression and the prognosis (*P* = 0.009). In conclusion, CD109 might be a CSC/CIC marker in epithelioid sarcoma. Moreover, CD109 is a promising prognostic biomarker and a molecular target of cancer therapy for sarcomas including ES.

## Introduction

Epithelioid sarcoma (ES) is a relatively rare and highly malignant soft tissue sarcoma (STS) accounting for <1% of all STSs [1]. The mainstay of treatment is aggressive, radical local resection or amputation. Currently other therapeutic options available for ES are limited. Therefore, a novel therapeutic option needs to be developed.

Recent studies have revealed that several human cancers contain a small subpopulation of cells called cancer stem-like cells (CSCs)/cancer initiating cells (CICs), which are defined by the ability of self-renewal, multi-differentiation potential, and tumorigenesis. Therefore, CSCs/CICs are believed to be responsible for the progression and relapse of cancer [2]. In the current study, we isolated CSCs/CICs based on aldehyde dehydrogenase 1 (ALDH1) activity. Human ALDHs are a family of NAD (P)+-dependent enzymes involved in detoxifying a wide variety of aldehydes to their corresponding weak carboxylic acids [3]. They serve to detoxify both xenobiotic aldehydes (eg. cyclophosphamide) and many other intracellular aldehydes, including ethanol and vitamin A [4]. Therefore, ALDH activity is important for drug resistance and the response to oxidative stress [5]. Recently ALDH1 activity was used, either alone or in combination with cell surface markers, to identify CSCs/CICs in hematologic malignancies and carcinomas derived from the lung and prostate [6-8].

We established a new ES cell line (designated ESX) from a 73-year-old woman. Next, we investigated CICs/CSCs in ES cell lines and isolated CSCs/CICs based on ALDH activity. Finally, we demonstrate that CD109 is a potential CSC/CIC marker that may be useful as a prognostic biomarker and a molecular target of cancer therapy for sarcomas, including ES. 

## Materials and Methods

### Ethics Statement

 Mice were maintained and experimented on in accordance with the guidelines of and after approval by the Ethics Committee of Sapporo Medical University School of Medicine, Animal Experimentation Center under permit number 08-006. Any animal found unhealthy or sick was promptly euthanized. All studies were approved by the Institutional Review Board of Sapporo Medical University Hospital. Written informed consent was obtained from all patients according to the guidelines of the Declaration of Helsinki.

### Primary tumor

 A 73-year-old Japanese woman was admitted to our hospital with a 9-month history of swelling of the left thigh. The swelling had gradually enlarged and become painful. A well-demarcated elastic soft mass was palpable in the medial aspect of the left thigh. Magnetic resonance imaging revealed a subcutaneous tumor and lymph node metastases in the inguinal region (Figure S1A). The tumor (3×3 cm) was homogeneously isointense relative to skeletal muscle in T1-weighted images, whereas it was heterogeneously iso- and hyperintense relative to skeletal muscle in T2-weighted images. Computed tomography revealed no pulmonary metastasis. The serum CA125 level was 6.6 U/ml (normal: <40 U/ml). Open biopsy showed that the tumor was composed of sheets of large cells with vesicular chromatin, prominent nucleoli, and amphophilic cytoplasm, with peripheral palisading of epithelioid cells around necrotic areas (Figure S1B). Immunohistochemical analysis revealed that the tumor was positive for AE1/AE3 and vimentin, but negative for CD34, CA125, and S-100. (Figure S1C). Although the tumor was weakly positive for INI1 analyzed by immunohistochemistry, fluorescence in situ hybridization (FISH) analysis revealed the heterozygous deletion of INI1 in 17 of 50 tumor cells (34%) (Figure S1D). Upon these findings, the tumor was diagnosed as proximal-type epithelioid sarcoma. Wide resection of the tumor and lymph node dissection were performed, but systemic chemotherapy was not. Unfortunately, pulmonary metastases developed 12 weeks after surgery and the patient died 16 weeks after the definitive surgery. 

### Establishment of a new ES cell line, ESX

 The resected specimen of the primary tumor was rinsed with phosphate-buffered saline, cut into small pieces with a scalpel and cultured in Iscove’s modified Dulbecco’s Eagle’s medium (IMDM; GIBCO BRL, Grand Island, NY) with 10% heat-inactivated fetal bovine serum (FBS; HyClone Laboratories, Inc., South Logan, UT). The tumors were incubated at 37°C in 5% CO_2_. The cell line (ESX) was maintained for more than 24 months. 

### Cell lines

 Human osteosarcoma cell lines (NY, U2OS and HOS), human Ewing sarcoma cell lines (SKES, WES, and RDES), the human synovial sarcoma cell line FUJI, and the human ES cell line VA-ES-BJ were purchased from the Japanese Collection of Research Bioresources Cell Bank (Tokyo, Japan) and American Type Culture Collection (Manassas, VA, USA). The human synovial sarcoma cell line YaFuSS and FU-EPS-1 were gifts from Dr J. Toguchida (Kyoto University) [9] and Dr H. Iwasaki (Fukuoka University) [10]. The human osteosarcoma cell line OS2000 and KIKU, and the human malignant fibrous histiocytoma cell lines MFH2003 and MFH2004 were established in our laboratory [11-14].

### ALDEFLUOR assay

 The ALDEFLUOR kit (StemCell Technologies, Vancouver, Canada) was used to separate the population with high ALDH1 activity. Cells (1×10^6^) were suspended in ALDEFLUOR assay buffer containing an ALDH1 substrate, bodipy-aminoacetaldehyde, at the concentration of 1μmol/L and incubated for 50 min at 37°C according to the manufacturer’s protocol. A specific inhibitor of ALDH1, diethylaminobenzaldehyde (DEAB), was used at 50mmol/L as a negative control. 

### CD109-positive cell sorting

 The cells were washed once with PBS and then centrifuged at 440g at 4°C for 5 min using an LX120 (Tomy, Tokyo, Japan). The cell pellets were resuspended and incubated for 60 min at 4°C with a 100-fold dilution of a mouse anti-CD109 antibody (R&D Systems). Then samples were washed with PBS 3 times and stained and incubated for 60 min at 4°C with a 500-fold dilution of an FITC-labeled anti-mouse secondary antibody (KPL, Gaithersburg, MD). Cell sorting was performed using a FACSAria II (BD Bioscience, San Jose, CA). Collected data were analyzed using BD FACSDiva V6.1.3 (BD Bioscience). Propidium isodide (PI; Life Technologies Corp.) was used to stain live cells. 

### RNA preparation and PCR analysis

 Total RNAs were extracted from cells using the RNeasyMini Kit (Qiagen, Hilden, Germany). cDNA was synthesized using Superscript III and an oligo(dT) primer (Life Technologies Corp.). Human Multiple Tissue cDNA Panels I and II, and the Human Fetal Multiple Tissue cDNA Panel (Clontech; Mountainview, CA) were used as normal tissue cDNAs. PCR was performed using KOD Dash (TOYOBO, Osaka, Japan) to detect CD109. The primer sequences used were 5’-TTGAATTCCCAATCCTGGAG-3’ and 5’-TTGTTGCCACTAACCACCAA-3’. The PCR mixture was denatured at 98°C for 2 min, followed by 30 cycles at 98°C for 15s, at 55°C for 2s, and at 74°C for 30s. GAPDH and beta-actin were used as internal controls. Real-time PCR was performed using the StepOne system (Life Technologies Corp.). Primers and probes were designed using the TapMan Gene expression assay (Life Technologies Corp.). Thermal cycling was performed with 40 cycles of 95°C for 1s, followed by 60°C for 20 min. Each experiment was done in triplicate and normalized to the GAPDH gene as an internal control.

### siRNA

 CD109 siRNA (siCD109) (5’-AAAGUUUGGACUCUGAUGACACCCA-3’) was designed using BLOCK-it RNAi (Life Technologies Corp.). Control siRNA was obtained from Life Technologies Corp. The siRNAs were transfected using Lipofectamine RNAiMAX transfection reagent (Life Technologies Corp.)

### Spherical colony formation assay

 Cells were plated at 1000 cells per well in six-well ultra-low attachment plates (Corning Inc., Corning, NY) and cultured in DMEM/F12 medium with 10ng/ml hEGF, 10ng/ml hbFGF, and 2% B-27 (Life Technologies Corp.) at 37°C in 5% CO_2_. On day 7, the number of colonies was counted under an inverted contrast microscope.

### Cell proliferation assay

 Cells were seeded in duplicate at a density of 2.5×10^4^ cells/well in 24-well plates. On the following day, siRNAs were transfected. At 48 hr, 72 hr and 120 hr cells were trypsinized and counted with a Coulter Counter (Beckman Coulter, Inc. Brea, CA). 

### Basement membrane matrix invasion assay

 Invasiveness was analyzed using a Matrigel™ Invasion Chamber (BD Biosciences). Briefly, 2.5×10^4^ cells were placed on inserts in the wells in IMDM with 10% FBS. After 52 hours, the cells were stained using a Hemacolor staining kit (Merck Millipore, Billerica, MA) and the degree of migration was determined. 

### Gene expression profiling

 RNA from ALDH^high^ cells was labeled with Cy5 dye and RNA from ALDH^low^ cells were labeled with Cy3 dye. The probe mixture was hybridized for 40 hours at 65°C using a Human Whole Genome Microarray (G4112F) (Agilent Technologies, Santa Clara, CA). The array was scanned after washing with a G2565BA Microarray Scanner and fluorescent signals were acquired using Feature Extraction software (Agilent Technologies). The average expression ratio of Cy5 to Cy3 was determined per gene. A dye swap experiment was also done to label ALDH^high^ and ALDH^low^ cells with Cy3 and Cy5, respectively. An average ratio of more than 2.0, reproducible in 2 experiments, was determined to indicate differential up-regulation in ALDH^high^ cells. The accession number of ArrayExpress is E-MEXP-3826. We focused on membrane protein-related genes for cell sorting and as therapeutic targets using antibodies. Therefore, we selected membrane protein-related genes based on information obtained from GeneCards (http://www.genecards.org) (Table S2), followed by screening of mRNA expression in ALDH^high^ and ALDH^low^ cells by RT-PCR (data not shown).

### Xenografting

 ALDH^high^ and ALDH^low^ cells freshly sorted from cell lines were washed and suspended in PBS. Then the cells (1×10^2^, 1×10^3^ and 1×10^4^ cells in 50μl of PBS) were mixed with an equal volume of Matrigel (BD Science) and subcutaneously injected into the bilateral sides of the lower back in female non-obese diabetic/severe combined immunodeficiency (NOD/SCID) mice (NOD.CB17-Prdkc^scid^/J, Charles River Laboratory, Yokohama, Japan). Tumor growth was monitored weekly for 10 weeks. Then the xenografted tumors were resected and analyzed.

### Immunohistochemical staining

 Formalin-fixed paraffin-embedded sections from 80 STS patients who underwent resection for stage I-III tumors and chemotherapy or radiotherapy for stage IV tumors between 2004 and 2009 in the Sapporo Medical University Hospital were used for CD109 staining as previously described [15]. The reactivity of the anti-CD109 antibody was determined by the staining pattern of the tumor cell membrane and graded as follows: 0 (no staining), 1 (partial staining of the membrane), 2 (mild to moderate circumferential staining of the membrane) and 3 (strong circumferential staining of the membrane). If the score was 2 or 3 in more than 10% of the tumor cells, it was considered to be positive. The clones used, antigen retrieval methods, and commercial sources of the antibodies used in the study are listed in Table S1. 

### Statistical methods

 The Mann-Whitney test was used to compare in vitro data and the differences in tumor volume using IBM SPSS Statistics (IBM Corp., Armonk, NY). The Fisher exact test was used to compare the associations between the CD109 expression level and clinicopathological factors using SAS software 9.3 (SAS Institute, Cary, NC). Postoperative disease-free survival (DFS) and overall survival (OS) were estimated using Kaplan-Meier plots. Prognostic significance was evaluated by the log-rank test. Univariate and multivariate analyses for hazards ratios (HR) in OS were performed by Cox’s proportional-hazards regression with backward selection using IBM SPSS Statistics. A probability of less than 0.05 was considered statistically significant. 

## Results

### Establishment of the ES cell line ESX

**Figure 1:**
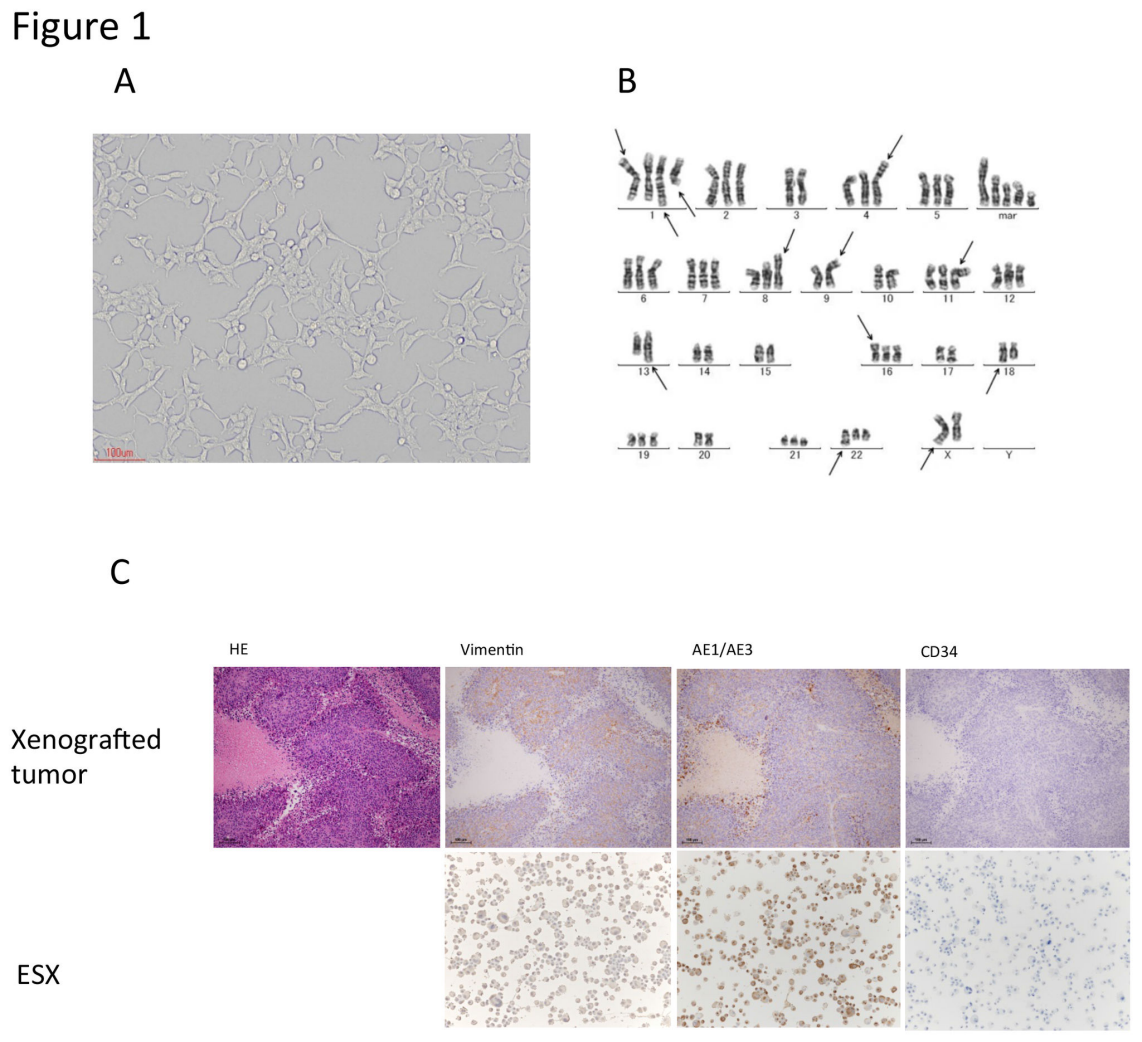
Establishment of the new epithelioid sarcoma cell line ESX. A. Phase-contact microscopy findings for ESX. B. Representative G-band karyotyping of ESX. The karyotype revealed 65~68, X, -X or –Y, add (X)(q22), +1, add(1) (p32), add(1)(q21), add(1)(q42), add(1)(q42), der(4;10)(q10;q10), add(8)(p11.2), -9, add(9)(p22), der(11)t(11;14)(p13;q13),-13, add(13)(q22), -14,-15,add(16)(p13.1), -17,-18, add(18)(q21),+21,add(22)(q13), +4~6mar[cp9]. Arrows indicate deletions and derivative chromosomes. C. Immunostaining analysis of the xenografted tumors (scale bar, 100μm) and ESX cell line for vimentin and AE1/AE3, and CD34 (original magnification ×100). Subcutaneous inoculations of ESX cells into NOD/SCID mice produced growing tumors. Histologically, the xenografted tumors consisted of a distinct nodular arrangement of the tumor cells, a tendency to undergo central degeneration and necrosis, and an epithelioid appearance with cytoplasmic eosinophilia. The atypical cells were positive for vimentin and AE1/AE3, but negative for CD34.

 A tumor cell culture obtained from a patient with ES of the left thigh (Figure S1) was maintained for over 1 year and designated ESX (Figure 1A). The ESX cells were spindle-shaped with large nuclei and grew as adherent, tightly packed monolayers, but had no epithelial cell morphology. The morphology was maintained across all cell passages. Karyotype analysis revealed massive rearrangement of chromosomes (Figure 1B). Immunohistochemical examination revealed that the atypical cells were positive for vimentin and AE1/AE3, but negative for CD34 (Figure 1C). Subcutaneous inoculations of ESX cells into NOD/SCID mice produced growing tumors. Histologically the xenografted tumors consisted of a distinct nodular arrangement of the tumor cells, a tendency to undergo central degeneration and necrosis, and an epithelioid appearance with cytoplasmic eosinophilia. Immunostaining analysis of the xenografted tumors also revealed a staining pattern similar to that of the ESX cells (Figure 1C) and the original tumor (Figure S1C). These findings indicated that the established cell line was consistent with the profile of the original tumor. The characteristics of ESX were consistent with the profile of the highly malignant original tumor.

### Identification of an ALDH^high^ population in ES cell lines

**Figure 2:**
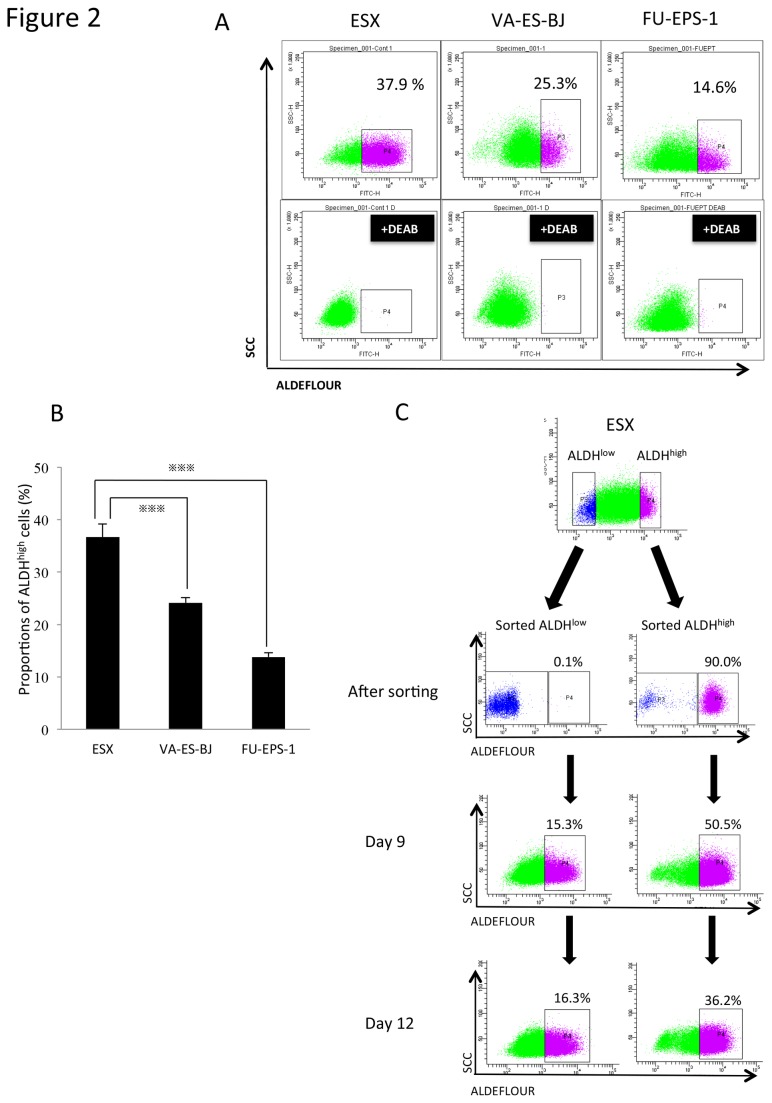
Identification of an ALDH^high^ population in ES cell lines. A. All 3 ES cell lines, ESX, VA-ES-BJ and FU-EPS-1, demonstrated ALDH activity. FACS analysis of ALDH1 expression in cells and the DEAB control. B. The proportions of ALDH^high^ cells in ESX, VA-ES-BJ and FU-EPS-1. Bars represent mean±SEM (n=4) of multiple experiments. ^※※※^ *p*<0.001, determined by the Mann-Whitney test. C. Differentiation of ALDH^high^ and ALDH^low^ cells in vitro. Sorted ALDH^high^ and ALDH^low^ cells were analyzed on days 0 (immediately after sorting), 9 and 12. Representative fluorescence-activated cell sorting analysis is shown.

 We performed ALDEFLUOR assay to detect ALDH^high^ populations containing CSCs/CICs in the epithelioid sarcoma cell lines. As shown in Figure 2A, all 3 ES cell lines (ESX, VA-ES-BJ, and FU-EPS-1) contained ALDH^high^ populations, although the proportion of ALDH^high^ cells varied. The mean proportions of ALDH^high^ cells were 36.6%, 14.2 % and 13.8% in ESX, VA-ES-BJ and FU-EPS-1, respectively. The proportions of ALDH^high^ cells in ES cell lines were higher than in the other sarcoma cell lines (Figure S2). The proportion of ALDH^high^ cells in ESX was significantly higher than in the others (*p*<0.001) (Figure 2B). We then analyzed the differentiation abilities of ALDH^high^ and ALDH^low^ ESX cells *in vitro* 9 days and 12 days after sorting (Figure 2C). ALDH^high^ and ALDH^low^ cells showed a tendency to differentiate into ALDH^low^ and ALDH^high^, respectively. These results could indicate the flexible plasticity of CSCs/CICs of ES cells. The frequency of ALDH^high^ cells converted from ALDH^low^ cells after in vitro culture was 16.3% on Day 12. On the other hand, when sorted ALDH^high^ cells were cultured in vitro the frequency of remaining ALDH^high^ cells was 36.2% on Day 12, indicating that ALDH^high^ cells could maintain higher enzyme activity than ALDH^low^ cells. 

### Cancer-initiating ability of ALDH^high^ cells of ES cell lines

**Figure 3:**
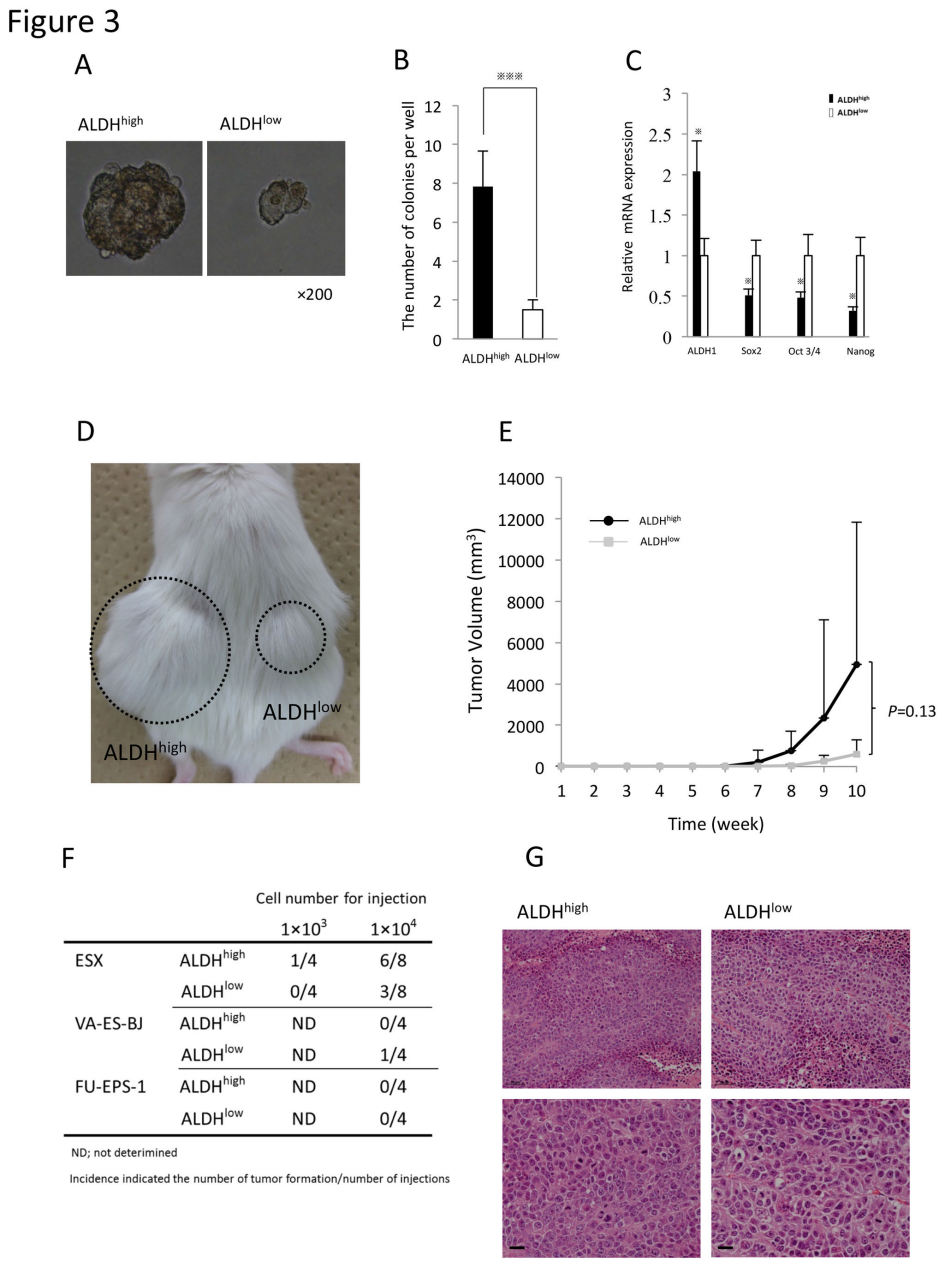
Cancer-initiating ability of ALDH^high^ cells *in vitro* and *in vivo*. A. The features of spherical colonies derived from resultant ALDH^high^ cells and ALDH^low^ cells of ESX. B. The number of spherical colonies from ALDH^high^ and ALDH^low^ cells of ESX. Bars represent mean±SEM (n=6). ^※※※^ *p*<0.001, determined by the Mann-Whitney test. C. The mRNA expression of stem/progenitor cell-related genes. RNA was isolated from freshly sorted spheroid cells (1x10^5^) on day 7. Bars represent mean±SEM. ^※^ *p*<0.05, determined by the Mann-Whitney test. D. The features of xenotransplanted cells *in* *vivo*. Macroscopic features of 1×10^4^ ALDH^high^ and ALDH^low^ cells of ESX in an NOD/SCID mouse at 10 weeks after xenotransplantation. E. Tumor growth curve of ALDH^high^ and ALDH^low^　cells of ESX. Bars represent the mean±SD (ALDH^high^ n= 4, ALDH^low^ n=2). F. Tumorigenesis of ALDH^high^ and ALDH^low^ cells of ES cell lines in NOD/SCID mice. G. H&E of the xenotransplanted tumors derived from ALDH^high^ and ALDH^low^cells of ESX (1×10^4^) (above; scale bar 50μm, below; 20μm).

 In a previous study, we showed that CSCs/CICs could generate floating spheroid-like bodies in a serum-free medium [16]. We therefore determined whether ALDH^high^ and ALDH^low^ cells of ESX could generate spherical colonies. As shown in Figure 3A, most ALDH^low^ cells died and the others formed a few small colonies. In contrast, the number of colonies derived from ALDH^high^ cells was significantly higher than that from ALDH^low^ cells (*p*<0.001) (Figure 3B). Next, we examined the expression of stem/progenitor cell-related genes *Sox2*, *Oct3/4, and Nanog* [17,18]. Interestingly, in the ALDH^high^ population the mRNA expression of *Sox2*, *Oct3/4*, and *Nanog* was lower than in ALDH^low^ (Figure 3C). These findings were in marked contrast to the CSCs/CICs of carcinomas, which suggested that gene expression of stem cell-related genes was not involved in the cancer-initiating ability, at least in epithelioid sarcoma [19]. In the ALDH^high^ cells of VA-ES-BJ and FU-EPS-1, the mRNA expression of stem/progenitor cell-related genes was not lower than in ALDH^low^ cells (Figure S3).

 To determine whether CSCs/CICs were abundant in the ALDH^high^ population, we performed xenograft transplantation of ALDH^high^ and ALDH^low^ cells of ESX, VA-ES-BJ, and FU-EPS-1 into NOD/SCID mice. The sorted ALDH^high^ and ALDH^low^ cells were injected subcutaneously into mice, and tumor growth was monitored weekly for 10 weeks. In ESX, 1×10^3^ ALDH^high^ cells showed tumorigenicity in one of four mice. In contrast, 1×10^3^ of ALDH^low^ cells failed to form tumors. On the other hand, 1×10^4^ ALDH^high^ and ALDH^low^ cells both showed tumorigenicity (Figure 3, D and E). However, the frequency of tumor formation was lower for ALDH^low^ cells than for ALDH^high^ cells (Figure 3F). The histology of the tumors derived from ALDH^high^ and ALDH^low^ cells of ESX showed no major differences (Figure 3G). In the case of FU-EPS-1, neither ALDH^high^ nor ALDH^low^ cells could form tumors. In VA-ES-BJ, ALDH^low^ cells formed a tumor but ALDH^high^ cells did not (Figure 3F). These findings suggested that ALDH^high^ cells of ESX contained a higher number of tumorigenic cells compatible with CSCs/CICs than ALDH^low^ cells. In contrast, we considered that VA-ES-BJ and FU-EPS-1 did not contain a CSCs/CICs population in ALDH^high^ cells that was similar to that of ESX. Therefore, ESX was used for the further characterization of the ALDH^high^ population containing CSCs/CICs. 

**Figure 4:**
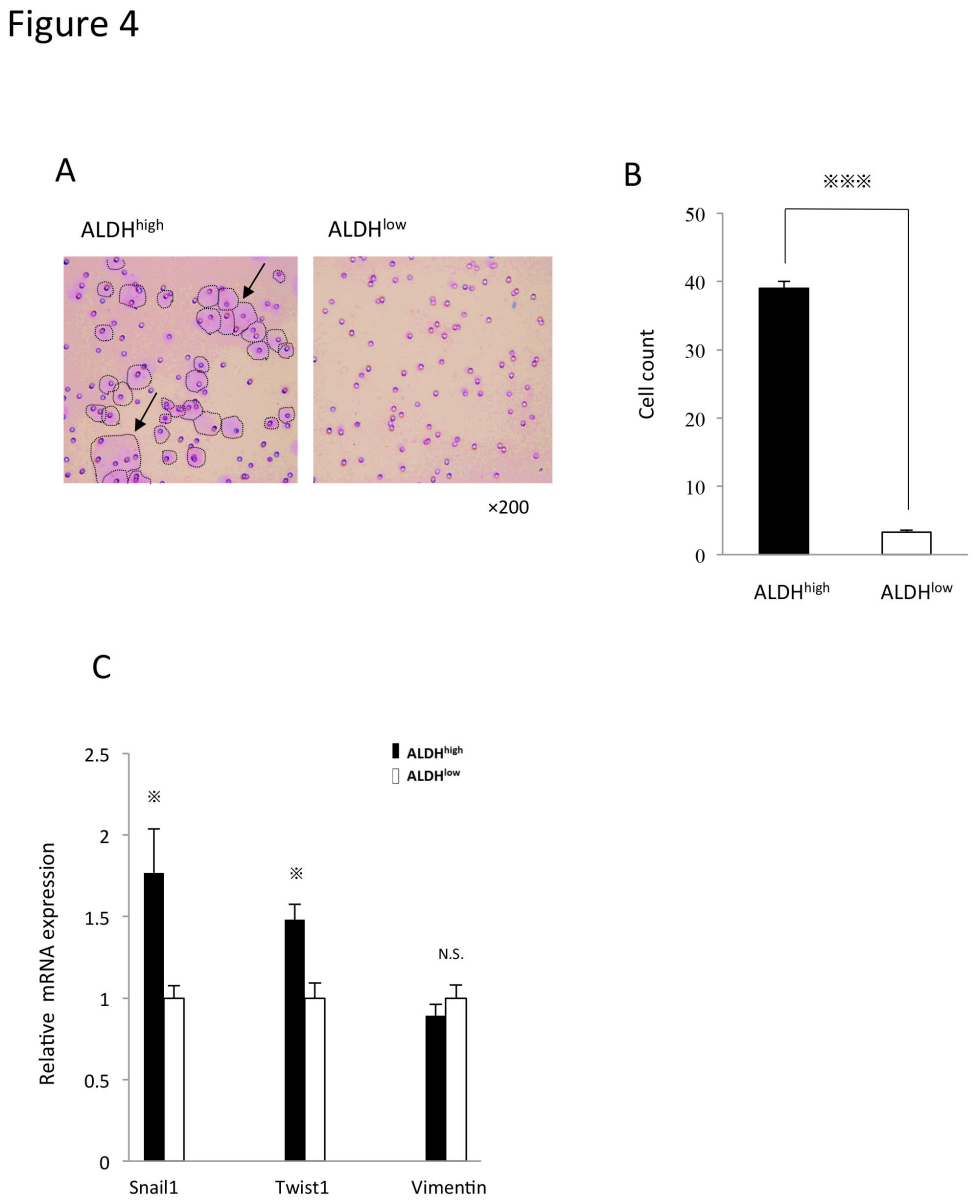
Invasive ability of ALDH^high^ cells of ESX. A. The features of invasion derived from resultant ALDH^high^ and ALDH^low^ cells. Invading cells are indicated by circular dots and arrows. B. The number of invasive cells. Bars represent mean±SEM (n=4). ^※※※^ *p*<0.001, determined by the Mann-Whitney test. C. The mRNA expression of EMT-related genes. Bars represent mean±SEM. ^※^ *p*<0.05, determined by the Mann-Whitney test.

 To examine the invasive potential of ALDH^high^ and ALDH^low^ cells in ESX, we performed in vitro basement membrane matrix invasion assay. The microscopic features of the invading cells of ALDH^high^ and ALDH^low^ are shown in Figure 4A. The number of invading cells for ALDH^high^ was significantly higher than that for ALDH^low^ cells (P<0.001) (Figure 4B). Furthermore, the mRNA expression of epithelial-mesenchymal transition (EMT)-related genes *Snail1 and Twist1* was upregulated in ALDH^high^ cells, supporting the invasive ability of the ALDH^high^ population (Figure 4C), which was compatible with the characteristics of CSCs/CICs. 

### Identification of the novel marker CD109 for the CSCs/CICs of ES

**Figure 5:**
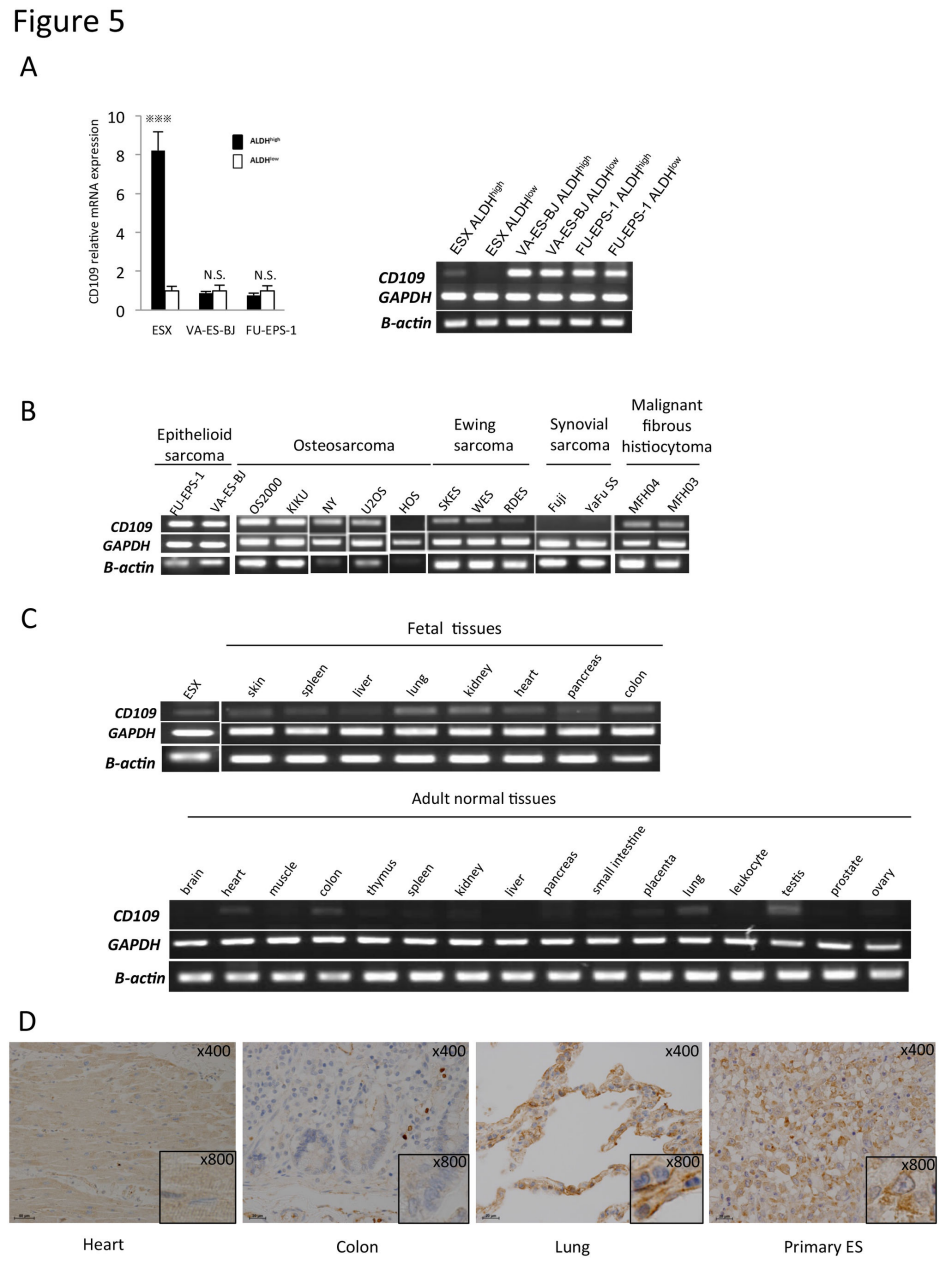
CD109 expression in sarcoma cells and normal adult and fetal tissues. A. Expression of CD109 mRNA in ES cell lines. Bars represent mean±SEM. ^※※※^ *p*<0.001, determined by the Mann-Whitney test. N.S.: not significant. B. Expression of CD109 mRNA in human sarcoma cell lines. Cell lines of epithelioid sarcoma (FU-EPS-1 and VA-ES-BJ), osteosarcoma (OS2000, KIKU, NY, U2OS, Saos-2, HuO9 and HOS), Ewing sarcoma (SKES, WES and RDES), synovial sarcoma (Fuji and YaFuSS) and malignant fibrous histiocytoma (MFH2003 and MFH2004) were used. C. Expression of CD109 mRNA in human fetal tissues (upper panel) and human adult tissues (lower panel). ESX was used as a positive control. D. Immunohistochemistry of CD109 in normal adult tissues.

 We screened the upregulated genes in ALDH^high^ cells using a cDNA microarray to identify membrane protein-related genes for cell sorting and therapeutic targets using antibodies. The upregulated membrane protein-related genes in ALDH^high^ cells of ESX are summarized in Table S2. Among them, we selected CD109, which was upregulated in ALDH^high^ cells, as a representative marker (Figure 5A). The other molecules listed in Table S2 were not upregulated in ALDH^high^ cells assessed by RT-PCR (data not shown). CD109, a GPI-anchored glycoprotein, was originally identified as a leukemia antigen. It has been reported that CD109 is expressed on activated T lymphocytes and platelets, endothelial cells and a subpopulation of CD34+ hematopoietic stem and progenitor cells [20,21]. CD109 has also been reported as the cell surface antigen on acute myeloid leukemia [20]. On the other hand, in the other ES cell lines there was no difference of CD109 mRNA expression between ALDH^high^ and ALDH^low^ cells. These results supported the hypothesis that CD109 could be essential to maintain the cancer-initiating ability of CSCs/CICs in ALDH^high^ cells of ESX. 

 We assessed the CD109 mRNA expression profiles in sarcomas and human fetal and normal adult tissues by RT-PCR. In addition to ES, CD109 mRNA expression was observed in 9 of 14 (64%) sarcoma cell lines (Figure 5B). As shown in Figure 5C, CD109 mRNA was weakly expressed in fetal skin, spleen, liver, lung, kidney, heart, pancreas and colon tissues. In adult tissues, CD109 mRNA was also weakly expressed in the lung, heart, small intestine, and testis. However, CD109 protein was expressed in alveoli of the lung, but not in heart and colon tissues (Figure 5D). These results suggested that CD109 could be an antigen highly expressed in various human sarcomas.

### CD109 is associated with cancer-initiating ability and TGFβ/Smad signaling

**Figure 6:**
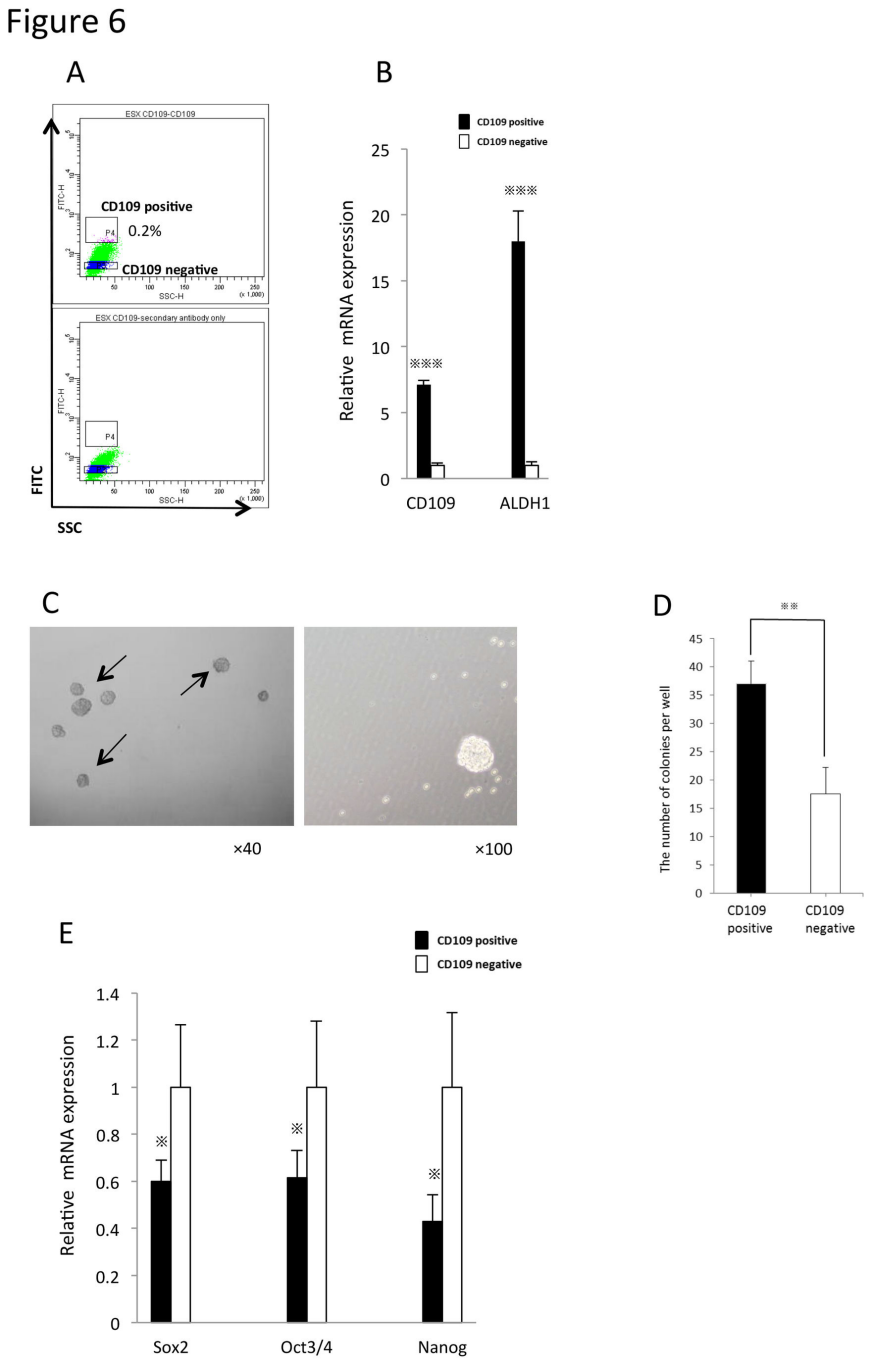
CD109 is a representative marker for the features of cancer-initiating ability. A. FACS analysis of CD109 positive cells in ESX was shown. B. The mRNA expression of CD109 and ALDH1A1. Bars represent mean±SEM. ^※※※^ *p*<0.001, determined by the Mann-Whitney test. C. The features of spherical colonies (indicated by arrows) derived from resultant CD109-positive cells and CD109-negative cells of ESX (original magnification ×40). D. The number of spherical colonies from CD109-positive cells and CD109-negative cells of ESX. Bars represent mean±SEM (n=3). ^※※^ *p*<0.01, determined by the Mann-Whitney test. E. The mRNA expression of stem/progenitor cell-related genes. Bars represent mean±SEM. ^※^ *p*<0.05, determined by the Mann-Whitney test.

 The proportion of CD109-positive cells was 0.2% in ESX (Figure 6A), which was lower than that of ALDH^high^ cells (Figure 1A). However, higher expression of ALDH1 mRNA was detected in CD109-positive cells than in CD109-negative cells (Figure 6B). Moreover, the number of spherical colonies derived from CD109-positive cells was higher than that from CD109-negative cells (Figure 6, C and D). In addition, the expression of stemness-related genes was lower in CD109-positive cells than in CD109-negative cells (Figure 6E). These features of CD109-positive cells were similar to those of ALDH^high^ cells and suggested that CD109 might regulate cancer-initiating ability in ESX.

**Figure 7:**
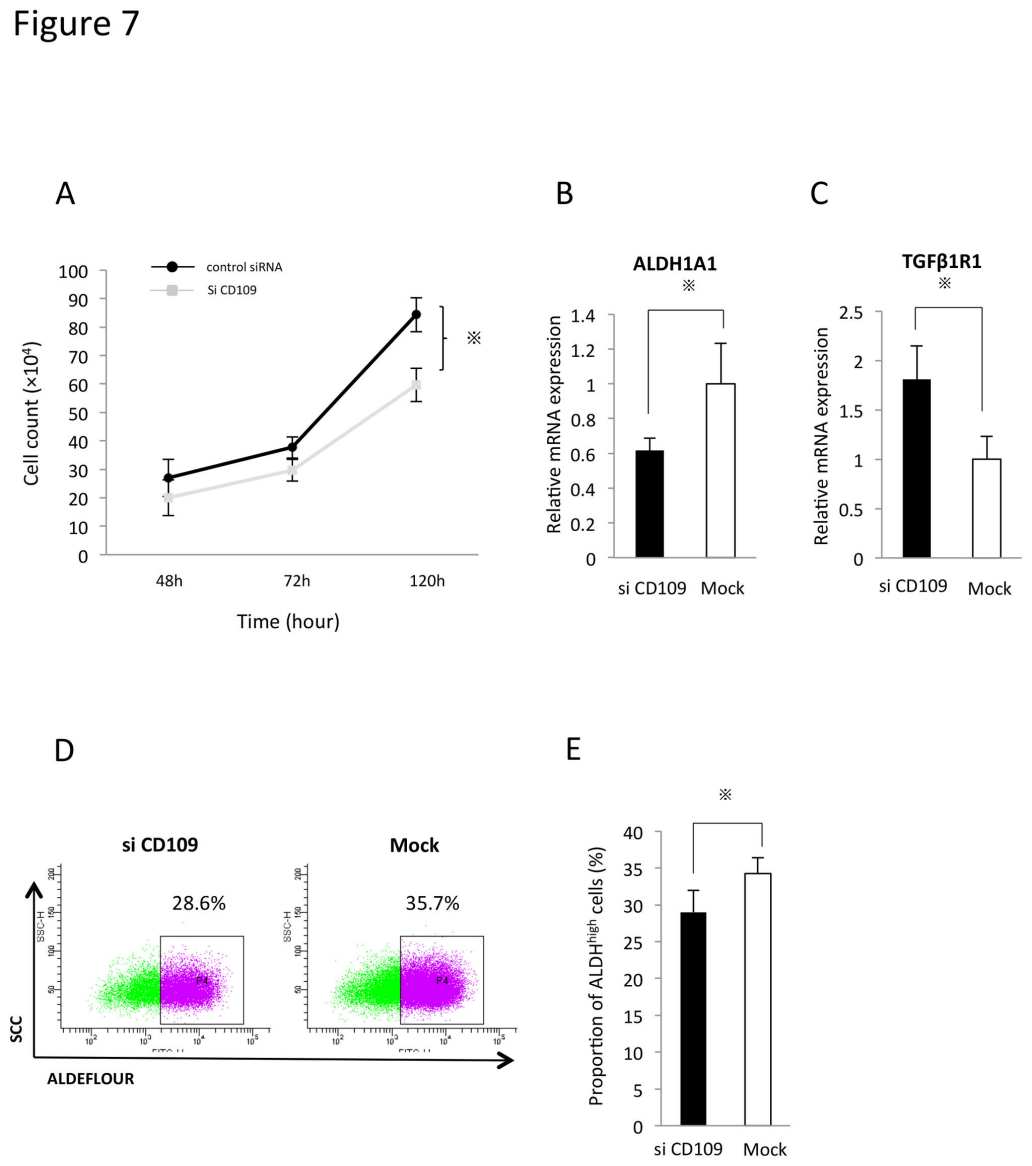
CD109 positively regulates ALDH activity and negatively regulates the TGFβ/Smad signaling pathway. A. The cell proliferation curve of ESX treated with SiCD109 or control siRNA. Bars represent mean±SEM (n=4). ^※^ *p*<0.05, determined by the Mann-Whitney test. B, C. mRNA expression of ALDH1A1 (D) and TGFβ1R1 (E). The expression of ALDH1A1 and TGFβ1R1 was evaluated by real-time PCR two days after transfection. D. FACS analysis of ALDH activity. E. The proportion of ALDH^high^ cells. Bars represent mean±SEM (n=4). ^※^ *p*<0.05, determined by the Mann-Whitney test.

 Next, we examined the effect of CD109 knockdown on cell proliferation. ESX cells were treated with siCD109, trypsinized and counted after 48 hr, 72 hr and 120 hr. Expression of both mRNA and protein of CD109 was downregulated (Figure S4, A, B and C). As shown in Figure 7A, siCD109 significantly inhibited cell proliferation (*p*<0.05). These results also supported the idea that CD109 plays an important role in cancer-initiating ability. 

 CD109 is a component of the TGF-β1 receptor 1 complex and negatively regulates TGF-β/Smad signaling [22]. Therefore, we hypothesized that CD109 could positively regulate ALDH1 activity and negatively regulate TGFβ1R1 expression in the TGF-β/Smad signaling pathway. We examined whether downregulation of ALDH1A1 and upregulation of TGFβ1R1 were induced by silencing of CD109. As shown in Figure 7B, ALDH1A1 mRNA was downregulated in siCD109-transfected ESX cells. On the other hand, TGFβ1R1 mRNA was upregulated in the siCD109-transfected cells (Figure 7C). Moreover, silencing of CD109 reduced the ALDH^high^ population in ESX cells (Figure 7, D and E). These findings suggested that CD109 positively regulated ALDH1A1 activity and negatively regulated the TGF-β/Smad signaling pathway. 

### CD109 protein is associated with poor prognosis in soft tissue sarcoma patients

**Figure 8:**
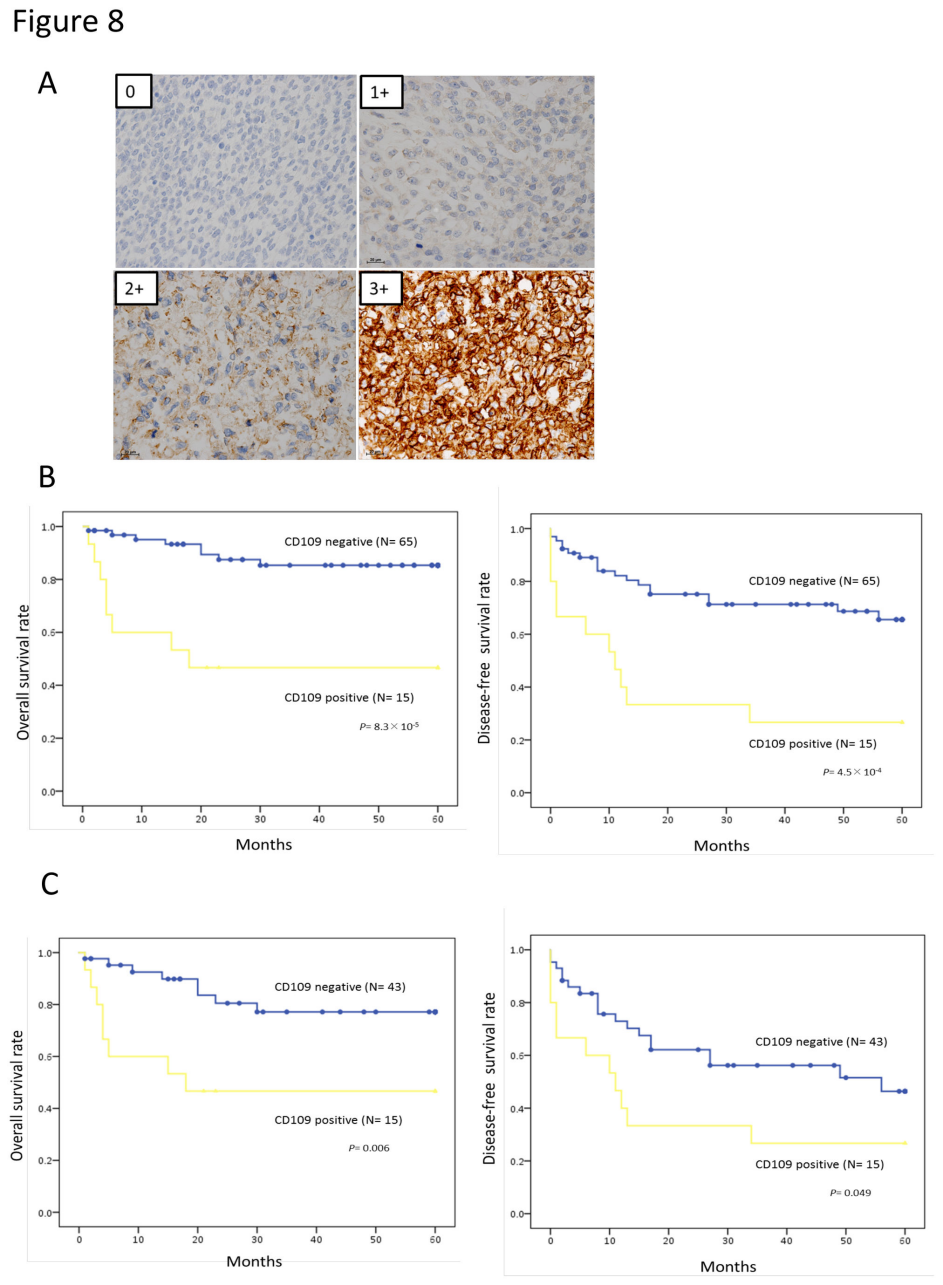
Expression of CD109 protein is associated with poor prognosis in soft tissue sarcoma patients. A. Representative immunostaining of CD109 (original magnification x400). B, C. Prognosis estimated by Kaplan-Meier plots for patients with soft tissue sarcoma including (B) and excluding (C) the patients with well-differentiated liposarcoma.

 To determine the clinical relevance of CD109 expression in soft tissue sarcomas (STS), we evaluated CD109 expression by immunohistochemistry in the primary extremity lesions of 80 STSs. The clinical characteristics of these patients are summarized in Table S3. Representative staining patterns with the anti-CD109 antibody are shown in Figure 8A. Positive expression of CD109 protein was identified in 18% (15/80) of the STSs. Higher expression of CD109 was significantly associated with histologic grade, tumor stage, and distant metastases (p=0.021, 0.0012, and 0.0003 respectively). However, no other significant correlation was found between CD109 expression and other clinical parameters of the STSs. As shown in Figure 8B and 8C, positive CD109 expression, including in well-differentiated liposarcomas, was significantly associated with decreased probabilities of overall survival (OS) and disease-free survival (DFS) (P=8.3×10^-5^　and 4.5×10^-4^, respectively). Moreover, excluding well-differentiated liposarcomas, positive CD109 expression was also significantly associated with decreased probabilities of OS and DFS (P=0.006 and 0.049, respectively). The OS rates at 5 years were 46.7% for CD109-positive patients and 85.3% in those who were CD109 negative. Several variables were tested to assess whether they had an impact on survival. Univariate and multivariate analyses revealed that the histologic grade and expression of CD109 were independent risk factors for poor outcome (Table 1, Table 2). The hazard ratio (HR) of OS for the CD109-positive group was 3.85 (95% confidence interval CI, 1.40-10.55). Thus, CD109 might be a predictive biomarker for both distant metastasis and the prognosis of STSs in the clinical setting. 

**Table 1:** Univariate analysis of the variables in overall survival.

Variable	Hazard ratio	95% CI	*P* value
Tumor size			
<5cm	1		
5-10cm	3.38	0.70-16.2	0.129
>10cm	2.21	0.46-10.6	0.321
Tumor depth			
Superficial	1		
Deep	24.0	0.024-24154	0.367
Histologic grade			
I	1		
II	3.18	0.28-35.1	0.344
III	20.4	2.66-157	0.004
CD109 expression			
Negative	1		
Positive	5.72	2.13-15.3	0.001

CI: confidence interval

**Table 2:** Multivariate analysis of the variables in overall survival.

Variable	Hazard ratio	95% CI	*P* value
Histologic grade			
I	1		
II	2.03	0.177-23.39	0.570
III	13.9	1.75-111.7	0.013
CD109 expression			
Negative	1		
Positive	3.85	1.40-10.55	0.009

CI: confidence interval

## Discussion

 In the present study, we (i) established and characterized the new ES cell line ESX; (ii) demonstrated that the ALDH^high^ population of ESX contained CSCs/CICs showing *in vitro and in vivo* tumorigenesis; (iii) found that high expression of CD109 in ALDH^high^ cells was important to maintain tumorigenesis as the important feature of CSCs/CICs; and (iv) showed the prognostic impact of CD109 expression on patients with STSs. ES is a rare, slow-growing malignant tumor. With an infiltrative growth pattern and a propensity for extension along fascial planes, and nerves, it is characteristically associated with multiple local recurrences and late metastasis [23]. The effects of multiagent chemotherapy and radiotherapy remain unclear; therefore, novel therapeutic options need to be developed. The establishment of an experimental model is imperative to investigate the biological characteristics of ES and develop novel therapeutic options. To the best of our knowledge, only 11 human ES cell lines have been reported to date [10,24-33]. ESX might therefore facilitate further studies on the biological characteristics of this rare tumor entity. 

Using ESX, we tried to identify CSCs/CICs of ES. Previously, we performed side population (SP) analysis to identify CSCs/CICs of sarcomas [16]. However, SP cells were hardly detected in ESX (data not shown). Therefore, we used the ALDEFLUOR assay. In this study, we demonstrated that an ALDH^high^ population existed in ESX, and that the ALDH^high^ cells possessed repopulating capacity and high tumor-forming ability *in vitro and in vivo*. ALDH is a cytosolic isoenzyme involved in the detoxification of intracellular aldehydes by oxidation and conversion of retinol to retinoic acid, and it confers resistance to chemotherapeutic agents such as cyclophosphamide [34]. Therefore, it makes sense that ALDH^high^ cells contain CSCs/CICs. Visus et al. were the first to describe the use of ALDH1A1 as a putative CSC/CIC marker in head and neck squamous cell carcinomas [35]. In the field of sarcoma, Awad et al. tested ALDH activity in Ewing sarcoma, defining CSCs/CICs as those cells that showed the highest ALDH activity [36]. However, there are no other reports regarding CSCs/CICs of sarcoma. This is the first report identifying and characterizing of CSCs/CICs of ES. The proportion of ALDH^high^ cells in ES cell lines was clearly higher than in the other sarcoma cell lines (Figure S2). These results were compatible with the chemotherapy-resistant characteristics of ES. 

Expression of the stem cell-related genes *Sox2*, *Oct3/4*, and *Nanog* is also used to characterize CSCs/CICs [37]. These are essential for the maintenance of pluripotent embryonic stem cells and germ cells, as well as CSCs/CICs [38]. Twist and Snail, which could promote EMT, are also considered to be key factors in the maintenance of CSCs/CICs. Twist and Snail induce not only increased potential for invasiveness and metastases, but also increased ability to form spheres and generate tumors in xenografts [39]. In this study, the ALDH^high^ population expressed *Twist 1 and Snail 1* at higher levels than ALDH^low^ cells. ALDH^high^ cells of ESX had higher invasive ability, which was considered to be compatible with previous reports [7,8]. On the other hand, expression of *Sox2, Oct3/4* and *Nanog* was lower in ALDH^high^ cells. The reason for this discrepancy in the expression status between ALDH^high^ and ALDH^low^ cells in the case of ESX remains unknown. Velcheti et al. reported that high expression of Sox2 was correlated with good prognosis in patients with non-small cell lung carcinomas [40]. These results suggested that Sox2 was not necessarily related to tumorigenicity and malignant potential. Alternatively, we speculate that CSCs/CICs of ES might have more differentiated characteristics with downregulation of Sox2 than those of carcinomas.

Using a cDNA microarray, we found that CD109 was upregulated in ALDH^high^ cells. CD109, a GPI-anchored glycoprotein, was originally identified as a leukemia antigen. It has been reported that CD109 is expressed on activated T lymphocytes and platelets, endothelial cells and a subpopulation of CD34+ hematopoietic stem and progenitor cells [20,21,41]. CD109 is also expressed in keratinocytes and contributes to the inhibition of extracellular matrix production in scleroderma [42]. In addition, it is also expressed in malignancies of the lung, esophagus, cervix, urinary tract and breast and plays a role in the tumor growth of oral cancer [43]. The present study also demonstrated that CD109 was highly expressed in sarcoma but not in normal tissues. The expression status suggested that CD109 might be a candidate therapeutic target not only for sarcoma but also for epithelial cancer. 

CD109 is a TGF-β co-receptor, a component of the TGF-β1 receptor 1 (TGFβ1R1) complex. It accelerates TGF-β receptor degradation and negatively regulates TGF-β/Smad signaling [44,45]. In some human cancers, CD109 actually impairs TGF-β/Smad signaling [46]. TGF-β can play both tumor-suppressive and tumor-promoting roles. Especially in the early phase of cancer initiation, TGF-β acts as an anti-oncogenic factor [47]. In this study, ALDH^high^ cells of ESX showed higher tumorigenicity and higher expression of CD109. In addition, silencing of CD109 upregulated TGFβ1R1 mRNA in ESX cells. These findings suggested that CD109 expressed in ALDH^high^ cells of ESX promoted cancer-initiating ability as the result of the CD109-mediated inhibition of TGF-β. 

CD109-positive cells of ESX highly expressed ALDH1 and showed higher tumorigenicity than CD109-negative cells. Furthermore, the expression status of stemness-related genes of CD109-positive cells was similar to that of ALDH^high^ cells of ESX. Therefore, we considered that CD109 could be a representative molecule of ALDH^high^ cells. In addition, knockdown of CD109 decreased ALDH1 activity in ESX cells. These results suggested that CD109 might regulate ALDH1 activity and confer the characteristics of CSCs/CICs in ESX cells. However, the proportion of CD109-positive cells was lower than that of ALDH^high^ cells of ESX. Therefore we hypothesized that other factors might also regulate ALDH1 activity.

The evaluation of the clinical specimens of STSs revealed a strong correlation between CD109 expression and DFS and OS, suggesting that CD109 could be a promising prognostic biomarker in STSs. Although it is reported that CD109 is preferentially expressed in the early stage of tumorigenesis in oral tumor and urothelial carcinomas [43,46], we demonstrated that high expression of CD109 was significantly associated with advanced stage in STSs. These results suggest that the pathophysiological function of CD109 protein in sarcomas is different from that in carcinomas. Therefore, we speculate that CD109 expression in STSs is deeply involved in invasion and metastasis. 

In conclusion, we established the novel ES cell line ESX. Next, we investigated CICs/CSCs in ES cell lines and isolated CSCs/CICs based on ALDH activity. Finally, we demonstrated that CD109 is a potential CSC/CIC marker, prognostic factor and molecular target for STSs, including ES. 

## Supporting Information

Table S1 **List of commercial sources of the antibodies used in the study.**
(DOC)

Table S2 **List of the 37 membrane protein-related related upregulated (rate ≥2.0) genes in ALDH^high^ cells of ESX.**
(DOC)

Table S3 **Association between CD109 expression and clinical variables.**
(DOC)

Figure S1 **Clinical characteristics of the origin of the new epithelioid sarcoma cell line.** A. Magnetic resonance imaging reveals a subcutaneous tumor (3×3 cm) located in the left thigh (left panel, arrow) and lymph node metastases in the inguinal region (right panel, arrow).
B. H&E staining of the primary tumor from a resected specimen reveals typical features of epithelioid sarcoma, showing central necrosis with peripheral palisading of epithelioid cells around necrotic areas (scale bars, left 500μm, right 50μm).
C. Immunohistochemical analysis of the primary tumor for AE1/AE3, vimentin, CD34, CA125, S-100 and INI1 expression (scale bar, 50μm).
D. Fluorescence in situ hybridization (FISH) analysis using the INI1/CEP22 deletion probe performed according the protocol we previously described [48]. Heterozygous deletion demonstrated by the lack of one red signal (indicating the INI1 region) was detected (indicated by red arrow). Green signals indicate the centromeric region of chromosome 22.
(TIF)

Figure S2 **The proportions of ALDH^high^ cells in the sarcoma cell lines.** FACS analysis of ALDH1 activities of the cell lines of osteosarcoma (U2OS and OS2000), synovial sarcoma (Fuji and HS-SYII), Ewing sarcoma (WES and RD-ES) and malignant fibrous histiocytoma (MFH2003 and MFH2004) with and without DEAB control.
(TIF)

Figure S3 **The mRNA expression of stem/progenitor cell-related genes in epithelioid sarcoma cell lines, VA-ES-BNJ and FU-EPS-1.** RNA was isolated from freshly sorted spheroid cells (1x10^5^) on day 7. Bars represent mean±SEM. ^※^ *p*<0.05, determined by the Mann-Whitney test.
(TIF)

Figure S4 **CD109 knockdown using siRNA.** A. Real-time PCR analysis of CD109 mRNA expression of ESX after transfection of siCD109 or mock siRNA on day 2. Bars represent mean±SEM. ^※※※^ *p*<0.001 was determined by Student’s t-test.
B. Western blot analysis of siCD109 cells. Cell lysate with Nonident P-40 detergent solution was separated by sodium dodecylsulfate-polyacrylamide gel electrophoresis. Separated proteins were transferred onto polyvinylidene fluoride membranes and probed with a mouse anti-CD109 antibody (H-7; Santa Cruz Biotechnology, USA). β-Actin was used as a loading control. The anti-CD109 antibody was used at 100-fold dilution. The membrane was visualized with Chemiluminescent HRP Substrate (Millipore, Billerica, MA, USA) according to the manufacturer’s protocol and analyzed using Odyssey Fc Imaging System (LI-COR Biosciences, Lincoln, NE, USA). Anti-β-actin was used as an internal control.
C. Real-time PCR analysis of CD109 mRNA expression of ESX after transfection of siCD109 on days 3, 5, 7 and 10.
(TIF)
