# Supplementary material for: High Expression of CD109 Antigen Regulates the Phenotype of Cancer Stem-Like Cells/Cancer-Initiating Cells in the Novel Epithelioid Sarcoma Cell Line ESX and Is Related to Poor Prognosis of Soft Tissue Sarcoma
Source: PLoS One. 2013 Dec 20;8(12):e84187. doi: 10.1371/journal.pone.0084187 (PMC3869840; doi:10.1371/journal.pone.0084187)
Supplement: Table S1 — List of commercial sources of the antibodies used in the study. (DOC) [file pone.0084187.s001.doc]

**Table S1. List of commercial sources of the antibodies used in the study.**

| Antibody | Source | Dilution | Clone |
| --- | --- | --- | --- |
| AE1/AE3 | DAKO | No dilution | AE1/AE3 |
| Vimentin | DAKO | No dilution | V9 |
| CD34 | DAKO | No dilution | classII |
| S-100 | DAKO | No dilution | S-100 |
| CA125 | DAKO | No dilution | M11 |
| INI1(BAF47) | BD Transduction Laboratories | 1:50 | 25/BAF47 |
| CD109 | R ＆ D | 1:3000 | 496920 |
| CD109 | Santa Cruz | 1:100 | H-7 |
|  | |  |  |
