# Supplementary material for: High Expression of CD109 Antigen Regulates the Phenotype of Cancer Stem-Like Cells/Cancer-Initiating Cells in the Novel Epithelioid Sarcoma Cell Line ESX and Is Related to Poor Prognosis of Soft Tissue Sarcoma
Source: PLoS One. 2013 Dec 20;8(12):e84187. doi: 10.1371/journal.pone.0084187 (PMC3869840; doi:10.1371/journal.pone.0084187)
Supplement: Table S2 — List of the 37 membrane protein-related related upregulated (rate ≥2.0) genes in ALDHhigh cells of ESX. (DOC) [file pone.0084187.s002.doc]

**Table S2. List of the 37 membrane protein-related related upregulated (rate ≥2.0) genes in ALDH^high^** cells of ESX.

| **Rank** | **GeneName** | **SystematicName** | **Description** | **Ratio** |
| --- | --- | --- | --- | --- |
| 1 | PKMYT1 | NM_004203 | Homo sapiens protein kinase, membrane associated tyrosine/threonine 1 (PKMYT1), transcript variant 1, mRNA [NM_004203] | **9.56** |
| 2 | TMEM39A | NM_018266 | Homo sapiens transmembrane protein 39A (TMEM39A), mRNA [NM_018266] | **6.87** |
| 3 | TJP2 | NM_004817 | Homo sapiens tight junction protein 2 (zona occludens 2) (TJP2), transcript variant 1, mRNA [NM_004817] | **6.50** |
| 4 | TMEM49 | NM_030938 | Homo sapiens transmembrane protein 49 (TMEM49), mRNA [NM_030938] | **5.62** |
| 5 | STEAP1 | NM_012449 | Homo sapiens six transmembrane epithelial antigen of the prostate 1 (STEAP1), mRNA [NM_012449] | **5.60** |
| 6 | RIMS3 | NM_014747 | Homo sapiens regulating synaptic membrane exocytosis 3 (RIMS3), mRNA [NM_014747] | **5.42** |
| 7 | TMEM54 | NM_033504 | Homo sapiens transmembrane protein 54 (TMEM54), mRNA [NM_033504] | **5.34** |
| 8 | ERBB2 | NM_001005862 | Homo sapiens v-erb-b2 erythroblastic leukemia viral oncogene homolog 2, neuro/glioblastoma derived oncogene homolog (avian) (ERBB2), transcript variant 2, mRNA [NM_001005862] | **5.10** |
| 9 | AQP3 | NM_004925 | Homo sapiens aquaporin 3 (Gill blood group) (AQP3), mRNA [NM_004925] | **5.08** |
| 10 | EMP1 | NM_001423 | Homo sapiens epithelial membrane protein 1 (EMP1), mRNA [NM_001423] | **4.70** |
| 11 | CHRNA3 | NM_000743 | Homo sapiens cholinergic receptor, nicotinic, alpha 3 (CHRNA3), mRNA [NM_000743] | **3.78** |
| 12 | MPP3 | NM_001932 | Homo sapiens membrane protein, palmitoylated 3 (MAGUK p55 subfamily member 3) (MPP3), mRNA [NM_001932] | **3.39** |
| 13 | TBL1XR1 | NM_024665 | Homo sapiens transducin (beta)-like 1X-linked receptor 1 (TBL1XR1), mRNA [NM_024665] | **3.35** |
| 14 | ABCA5 | NM_018672 | Homo sapiens ATP-binding cassette, sub-family A (ABC1), member 5 (ABCA5), transcript variant 1, mRNA [NM_018672] | **3.30** |
| 15 | TMBIM4 | NM_016056 | Homo sapiens transmembrane BAX inhibitor motif containing 4 (TMBIM4), mRNA [NM_016056] | **3.25** |
| 16 | TNFRSF12A | NM_016639 | Homo sapiens tumor necrosis factor receptor superfamily, member 12A (TNFRSF12A), mRNA [NM_016639] | **3.04** |
| 17 | IGF1R | NM_000875 | Homo sapiens insulin-like growth factor 1 receptor (IGF1R), mRNA [NM_000875] | **3.01** |
| 18 | MAGEA6 | NM_175868 | Homo sapiens melanoma antigen family A, 6 (MAGEA6), transcript variant 2, mRNA [NM_175868] | **3.01** |
| 19 | TMEM62 | NM_024956 | Homo sapiens transmembrane protein 62 (TMEM62), mRNA [NM_024956] | **3.00** |
| 20 | RIPK5 | NM_015375 | Homo sapiens receptor interacting protein kinase 5 (RIPK5), transcript variant 1, mRNA [NM_015375] | **3.00** |
| 21 | TMED7 | NM_181836 | Homo sapiens transmembrane emp24 protein transport domain containing 7 (TMED7), mRNA [NM_181836] | **2.92** |
| 22 | TMEM130 | NM_152913 | Homo sapiens transmembrane protein 130 (TMEM130), mRNA [NM_152913] | **2.81** |
| 23 | TMEM113 | NM_025222 | Homo sapiens transmembrane protein 113 (TMEM113), mRNA [NM_025222] | **2.75** |
| 24 | OR7E24 | NR_002146 | Homo sapiens olfactory receptor, family 7, subfamily E, member 24 (OR7E24) on chromosome 19 [NR_002146] | **2.72** |
| 25 | EMP3 | NM_001425 | Homo sapiens epithelial membrane protein 3 (EMP3), mRNA [NM_001425] | **2.71** |
| 26 | TMEFF2 | NM_016192 | Homo sapiens transmembrane protein with EGF-like and two follistatin-like domains 2 (TMEFF2), mRNA [NM_016192] | **2.71** |
| 27 | TMEM60 | NM_032936 | Homo sapiens transmembrane protein 60 (TMEM60), mRNA [NM_032936] | **2.70** |
| 28 | GJB2 | NM_004004 | Homo sapiens gap junction protein, beta 2, 26kDa (connexin 26) (GJB2), mRNA [NM_004004] | **2.65** |
| 29 | TMEM41B | NM_015012 | Homo sapiens transmembrane protein 41B (TMEM41B), mRNA [NM_015012] | **2.57** |
| 30 | IL13RA1 | NM_001560 | Homo sapiens interleukin 13 receptor, alpha 1 (IL13RA1), mRNA [NM_001560] | **2.54** |
| 31 | MPP6 | NM_016447 | Homo sapiens membrane protein, palmitoylated 6 (MAGUK p55 subfamily member 6) (MPP6), mRNA [NM_016447] | **2.52** |
| 32 | TMEM32 | NM_173470 | Homo sapiens transmembrane protein 32 (TMEM32), mRNA [NM_173470] | **2.41** |
| 33 | EPHA4 | NM_004438 | Homo sapiens EPH receptor A4 (EPHA4), mRNA [NM_004438] | **2.39** |
| 34 | FZD10 | NM_007197 | Homo sapiens frizzled homolog 10 (Drosophila) (FZD10), mRNA [NM_007197] | **2.36** |
| 35 | CD109 | NM_133493 | Homo sapiens CD109 molecule (CD109), mRNA [NM_133493] | **2.31** |
| 36 | CD44 | NM_000610 | Homo sapiens CD44 molecule (Indian blood group) (CD44), transcript variant 1, mRNA [NM_000610] | **2.30** |
| 37 | MCAM | NM_006500 | Homo sapiens melanoma cell adhesion molecule (MCAM), mRNA [NM_006500] | **2.27** |
