# Supplementary material for: High Expression of CD109 Antigen Regulates the Phenotype of Cancer Stem-Like Cells/Cancer-Initiating Cells in the Novel Epithelioid Sarcoma Cell Line ESX and Is Related to Poor Prognosis of Soft Tissue Sarcoma
Source: PLoS One. 2013 Dec 20;8(12):e84187. doi: 10.1371/journal.pone.0084187 (PMC3869840; doi:10.1371/journal.pone.0084187)
Supplement: Table S3 — Association between CD109 expression and clinical variables. (DOC) [file pone.0084187.s003.doc]

**Table S**3. Association between CD109 expression and clinical variables.

| Variable | n | Positive | Negative | %positive | *P* value |
| --- | --- | --- | --- | --- | --- |
| Age (yrs) |  |  |  |  | 0.251 |
| <50 | 13 | 4 | 9 | 30 |  |
| >50 | 67 | 11 | 56 | 16 |  |
| Gender |  |  |  |  | 0.775 |
| Male | 38 | 8 | 30 | 21 |  |
| Female | 42 | 7 | 35 | 16 |  |
| Tumor depth |  |  |  |  |  |
| Superficial | 7 | 0 | 7 | 0 | 0.337 |
| Deep | 73 | 15 | 58 | 21 |  |
| Tumor size |  |  |  |  | 0.820 |
| <5cm | 21 | 3 | 18 | 12 |  |
| 5-10cm | 26 | 6 | 20 | 23 |  |
| >10cm | 33 | 6 | 27 | 18 |  |
| Histologic type |  |  |  |  | N.D. |
| Clear cell sarcoma | 1 | 1 | 0 | 100 |  |
| Dedifferentiated liposarcoma | 2 | 1 | 0 | 50 |  |
| Epithelioid sarcoma | 5 | 5 | 0 | 100 |  |
| Extraskeletal myxoid chondrosarcoma | 1 | 1 | 0 | 100 |  |
| Extraskeletal osteosarcoma | 2 | 0 | 2 | 0 |  |
| Leiomyosarcoma | 5 | 0 | 5 | 0 |  |
| Fibrosarcoma | 1 | 0 | 1 | 0 |  |
| MPNST | 1 | 0 | 1 | 0 |  |
| MFS | 20 | 2 | 18 | 10 |  |
| Pleomorphic MFH | 10 | 3 | 7 | 30 |  |
| Rhabdomyosarcoma | 1 | 0 | 1 | 0 |  |
| Synovial sarcoma | 5 | 1 | 4 | 20 |  |
| Myxoid liposarcoma | 4 | 1 | 3 | 25 |  |
| Well differentiated liposarcoma | 22 | 0 | 22 | 0 |  |
| Histologic grade |  |  |  |  | 0.013 |
| I | 30 | 1 | 29 | 3 |  |
| II | 20 | 5 | 15 | 25 |  |
| III | 30 | 9 | 21 | 30 |  |
| Stage |  |  |  |  | 0.0014 |
| I | 31 | 1 | 30 | 3 |  |
| II | 19 | 3 | 16 | 25 |  |
| III | 26 | 8 | 18 | 30 |  |
| IV | 4 | 3 | 1 | 75 |  |
| Distant metastasis |  |  |  |  | 0.0006 |
| Present | 27 | 11 | 16 | 40 |  |
| Absent | 53 | 4 | 49 | 8 |  |

MPNST: malignant peripheral sheath tummor; MFS: myxofibrosarcoma; MFH: malignant fibrous histiocytoma.
