# Supplementary figures and images for: High Expression of CD109 Antigen Regulates the Phenotype of Cancer Stem-Like Cells/Cancer-Initiating Cells in the Novel Epithelioid Sarcoma Cell Line ESX and Is Related to Poor Prognosis of Soft Tissue Sarcoma
Source: PLoS One. 2013 Dec 20;8(12):e84187. doi: 10.1371/journal.pone.0084187 (PMC3869840; doi:10.1371/journal.pone.0084187)

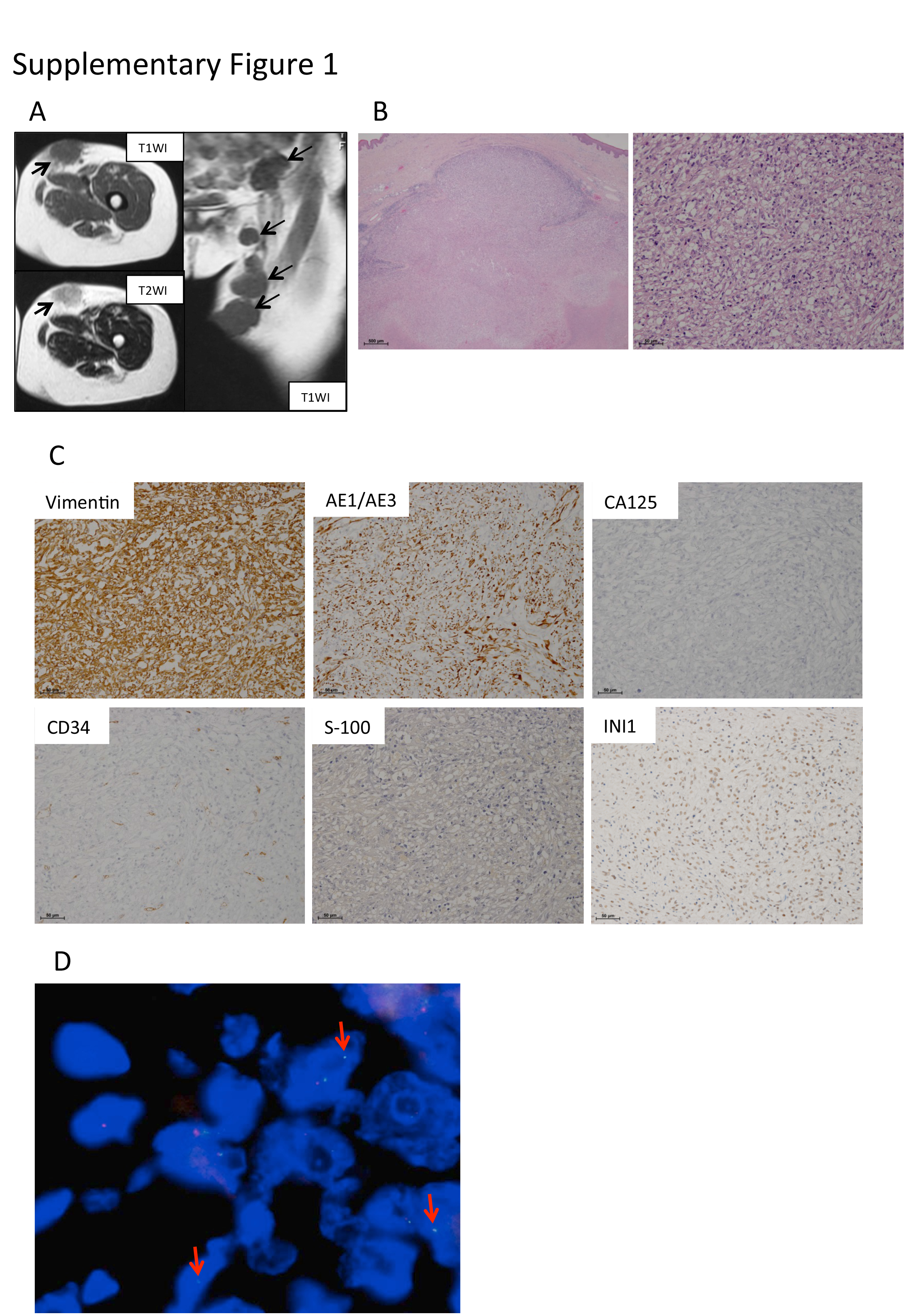

Supplement: Figure S1 — Clinical characteristics of the origin of the new epithelioid sarcoma cell line. A. Magnetic resonance imaging reveals a subcutaneous tumor (3×3 cm) located in the left thigh (left panel, arrow) and lymph node metastases in the inguinal region (right panel, arrow). B. H&E staining of the primary tumor from a resected specimen reveals typical features of epithelioid sarcoma, showing central necrosis with peripheral palisading of epithelioid cells around necrotic areas (scale bars, left 500μm, right 50μm). C. Immunohistochemical analysis of the primary tumor for AE1/AE3, vimentin, CD34, CA125, S-100 and INI1 expression (scale bar, 50μm). D. Fluorescence in situ hybridization (FISH) analysis using the INI1/CEP22 deletion probe performed according the protocol we previously described [48]. Heterozygous deletion demonstrated by the lack of one red signal (indicating the INI1 region) was detected (indicated by red arrow). Green signals indicate the centromeric region of chromosome 22. (TIF) [file pone.0084187.s004.tif]

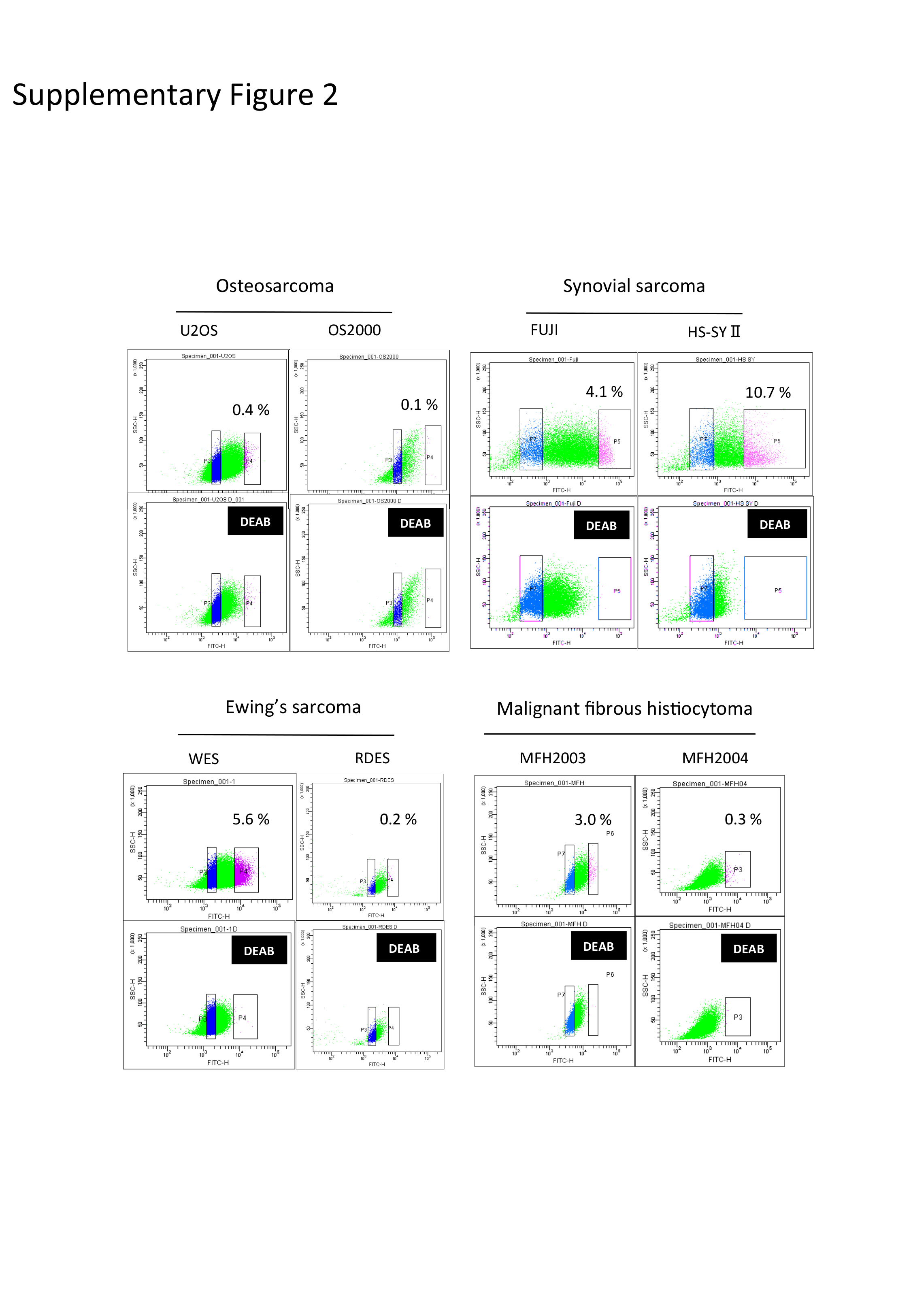

Supplement: Figure S2 — The proportions of ALDHhigh cells in the sarcoma cell lines. FACS analysis of ALDH1 activities of the cell lines of osteosarcoma (U2OS and OS2000), synovial sarcoma (Fuji and HS-SYII), Ewing sarcoma (WES and RD-ES) and malignant fibrous histiocytoma (MFH2003 and MFH2004) with and without DEAB control. (TIF) [file pone.0084187.s005.tif]

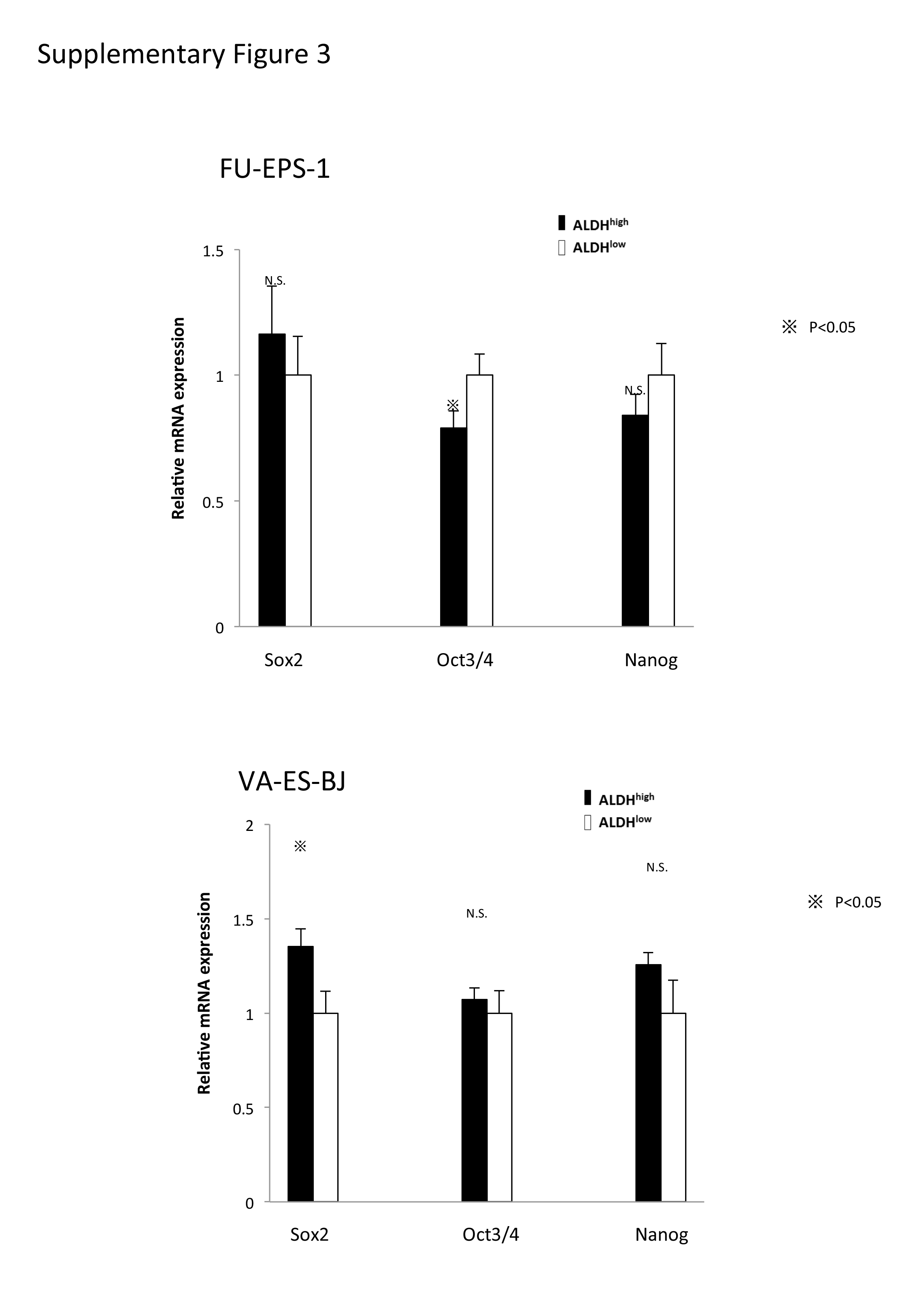

Supplement: Figure S3 — The mRNA expression of stem/progenitor cell-related genes in epithelioid sarcoma cell lines, VA-ES-BNJ and FU-EPS-1. RNA was isolated from freshly sorted spheroid cells (1x10^5^) on day 7. Bars represent mean±SEM. ※ p<0.05, determined by the Mann-Whitney test. (TIF) [file pone.0084187.s006.tif]

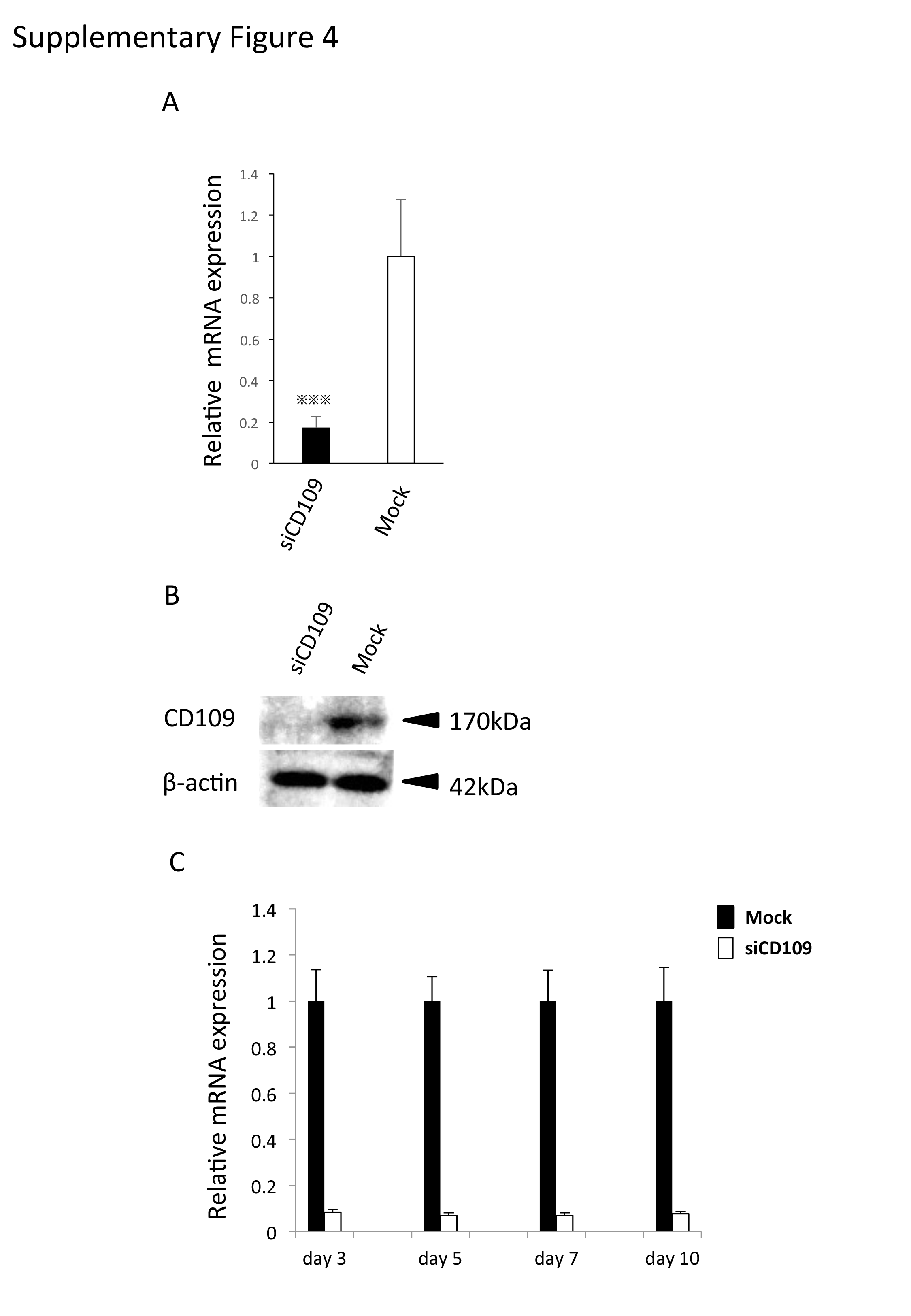

Supplement: Figure S4 — CD109 knockdown using siRNA. A. Real-time PCR analysis of CD109 mRNA expression of ESX after transfection of siCD109 or mock siRNA on day 2. Bars represent mean±SEM. ※※※ p<0.001 was determined by Student’s t-test. B. Western blot analysis of siCD109 cells. Cell lysate with Nonident P-40 detergent solution was separated by sodium dodecylsulfate-polyacrylamide gel electrophoresis. Separated proteins were transferred onto polyvinylidene fluoride membranes and probed with a mouse anti-CD109 antibody (H-7; Santa Cruz Biotechnology, USA). β-Actin was used as a loading control. The anti-CD109 antibody was used at 100-fold dilution. The membrane was visualized with Chemiluminescent HRP Substrate (Millipore, Billerica, MA, USA) according to the manufacturer’s protocol and analyzed using Odyssey Fc Imaging System (LI-COR Biosciences, Lincoln, NE, USA). Anti-β-actin was used as an internal control. C. Real-time PCR analysis of CD109 mRNA expression of ESX after transfection of siCD109 on days 3, 5, 7 and 10. (TIF) [file pone.0084187.s007.tif]
